# Evolutionary Genomics Reveals Multiple Functions of Arylalkylamine *N*-Acetyltransferase in Fish

**DOI:** 10.3389/fgene.2022.820442

**Published:** 2022-05-19

**Authors:** Yu Huang, Jia Li, Chao Bian, Ruihan Li, Xinxin You, Qiong Shi

**Affiliations:** ^1^ Shenzhen Key Lab of Marine Genomics, Guangdong Provincial Key Lab of Molecular Breeding in Marine Economic Animals, BGI Academy of Marine Sciences, BGI Marine, BGI, Shenzhen, China; ^2^ Department of Plant Biotechnology and Bioinformatics, Ghent University, VIB-Ugent Center for Plant Systems Biology, Ghent, Belgium; ^3^ BGI Education Center, College of Life Sciences, University of Chinese Academy of Sciences, Shenzhen, China

**Keywords:** melatonin biosynthesis, arylalkylamine *N*-acetyltransferase (AANAT), phylogeny, physiological function, fish

## Abstract

As an important hormone, melatonin participates in endocrine regulation of diverse functions in vertebrates. Its biosynthesis is catalyzed by four cascaded enzymes, among them, arylalkylamine *N*-acetyltransferase (AANAT) is the most critical one. Although only single *aanat* gene has been identified in most groups of vertebrates, researchers including us have determined that fish have the most diverse of *aanat* genes (*aanat1a*, *aanat1b,* and *aanat2*), playing various potential roles such as seasonal migration, amphibious aerial vision, and cave or deep-sea adaptation. With the rapid development of genome and transcriptome sequencing, more and more putative sequences of fish *aanat* genes are going to be available. Related phylogeny and functional investigations will enrich our understanding of AANAT functions in various fish species.

## Introduction

**FIGURE 1:**
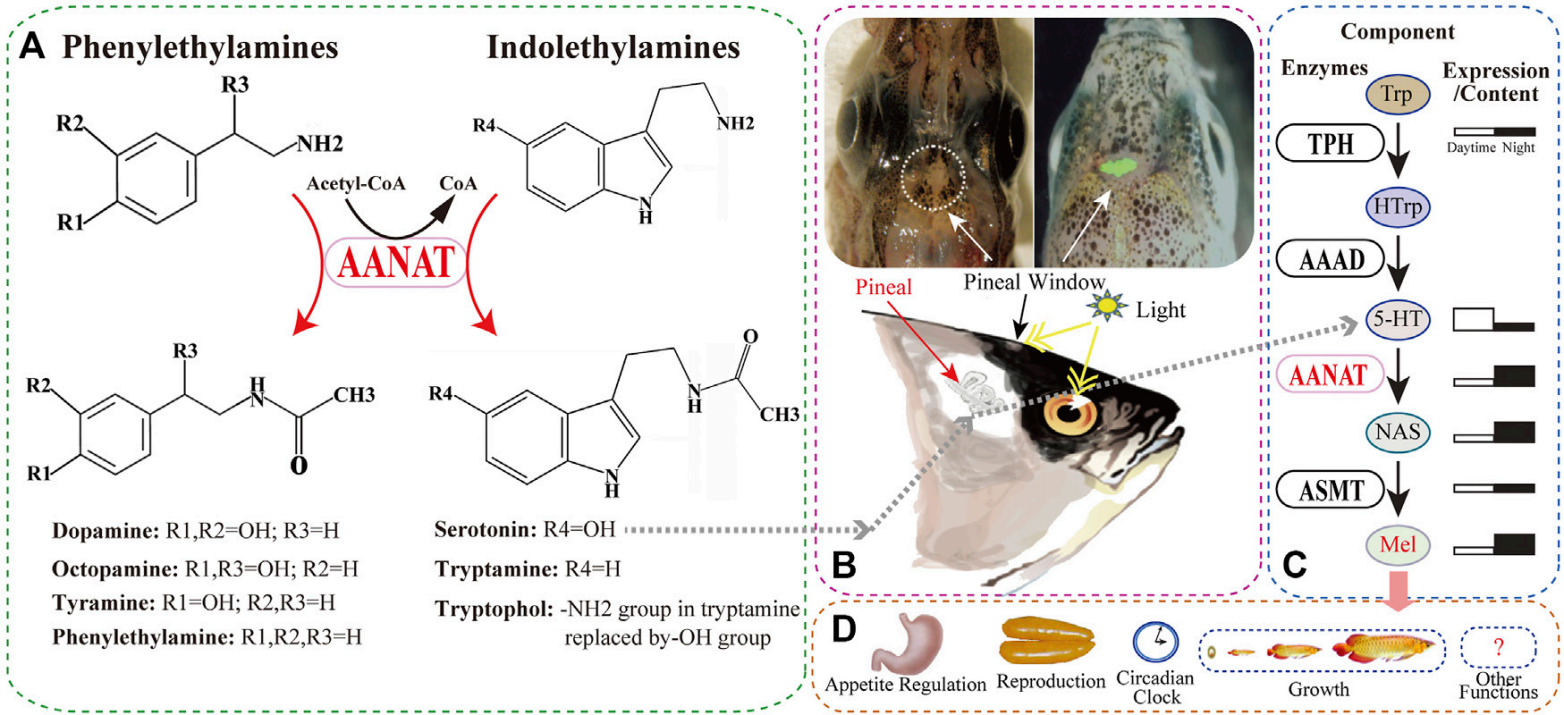
The substrates of AANAT and one of its products (melatonin; Mel) synthesized in fish. **(A)** AANAT can catalyze arylalkylamines, including phenylethylamines (dopamine, octopamine, tyramine, and phenylethylamine) and indolethylamines (serotonin and tryptamine). **(B)** The fish pineal organ. Upper left: the depigmented area around the pineal window (circle) of a polar cod (*Boreogadus saida*) head (adopted from Falcón et al., 2011). Upper right: zebrafish pineal gland with enhanced green fluorescent protein (EGFP) expressed under the control of *aanat2* promoter (Ben-Moshe et al., 2014). Down: melatonin synthesis in the fish pineal gland through regulation of light. **(C)** Melatonin biosynthesis pathway and related enzymes including AANAT. **(D)** Multiple important functions of melatonin in various fishes.

Arylalkylamine *N*-acetyltransferase (AANAT), also known as serotonin *N*-acetyltransferase (SNAT or NAT), belongs to the GCN5-related *N*-acetyltransferase (GNAT) superfamily. It is an acetyl-CoA-dependent enzyme (Wolf et al., 2002) and catalyzes the transfer of the acetyl group in acetyl-CoA to an arylalkylamine. The common AANAT arylalkylamine substrates are serotonin and dopamine (Figure 1A). AANATs play differential functions in various groups of organisms. In insects, multiple AANATs have evolved, particularly in mosquitoes (Han et al., 2012). Insect AANATs are mainly involved in cuticle formation, pigmentation, and some bioamine neurotransmitters and fatty acid amide metabolism, as well as circadian rhythms (O’Flynn et al., 2018; Zhang et al., 2019; Kamruzzaman et al., 2021; Zhang K. et al., 2021; Zhang et al., 2022). In vertebrates, AANATs play differential functions mainly through a small molecule, melatonin (*N*-acetyl-5-methoxytryptamine; Figure 1).

As one of the important products of the AANAT enzyme (Figure 1C), melatonin participates in the regulation of animal behaviors and physiology such as reproduction, growth, and immunity (Shi, 2005; Coon and Klein, 2006; Li et al., 2015; Kuz’mina, 2020). Initially isolated from bovine pineal gland (Lerner et al., 1958), melatonin was subsequently identified in humans (Brzezinski, 1997) and other vertebrates including fish (Esteban et al., 2013). It has also been detected in various taxa of invertebrates (Vivien-Roels and Pévet, 1993) and even in plants, fungi (Rodriguez-Naranjo et al., 2012), and bacteria (Tilden et al., 1997), although it may perform differential functions in various groups of organisms (Zhao et al., 2019).

Melatonin works as the “gear” of the biological clocks in animals, which are influenced by light/dark or temperature changes in a timescale of day (circadian rhythms) or year (circannual rhythms) (Zhao et al., 2019). In fish, the regulation of melatonin is usually through the eye and depigmented pineal window in response to light (Figure 1B). Therefore, melatonin was previously reported to be mainly synthesized in pinealocytes and then released into the bloodstream to reach central and peripheral organs for diverse roles. However, recent reports indicate that a majority of melatonin is synthesized in other tissues such as gut and skin rather than the pineal gland (Yasmin et al., 2021; Sevilla et al., 2022), suggesting that it can also regulate biological activities by binding to its specific receptors in target tissues (Shi et al., 2004; Isorna et al., 2009; Kuz’mina, 2020).

In fish and other animals, melatonin is synthesized from the precursor tryptophan (Trp) through four enzyme-catalyzed reactions (Figure 1C). The first step is catalyzed by tryptophan hydroxylase (TPH), converting Trp to 5-hydroxytryptophan (HTrp) (Xu et al., 2019), followed by the action of aromatic *L*-amino acid decarboxylase (AAAD), changing HTrp to serotonin (5-HT) (Li et al., 2018). The third step acetylates 5-HT to *N*-acetylserotonin (NAS) by arylalkylamine *N*-acetyltransferase (AANAT) (Li et al., 2015), and the final synthesis of melatonin is catalyzed by acetylserotonin-*O*-methyltransferase (ASMT) (Zhang et al., 2017).

During these four steps, AANAT is the penultimate enzyme in the melatonin biosynthesis pathway (Falcón et al., 2010) that is well conserved through evolution in various vertebrates (Li et al., 2015). It has a structurally conserved fold consisting of an eight-stranded *β* sheet flanked by five *α* helices, and also shares four conserved motifs designated A–D (Hickman et al., 1999), among which motif B contributes acidic residues to the serotonin binding slot (Wolf et al., 2002).

It has been previously reported that AANAT catalyzes the rate-limiting step in animal melatonin biosynthesis (Foulkes et al., 1996). However, many other reports strongly suggest that, rather than AANAT, the last enzyme ASMT might also control melatonin production (Liu and Borjigin, 2005; Chattoraj et al., 2009). Instead, AANAT should be regarded as the “melatonin rhythm-generating enzyme”, because its large nocturnal increase in activity drives the daily rhythm in melatonin secretion (Figure 1C) (Chattoraj et al., 2009). In specific, expression of *aanat* genes is driven directly by the circadian clock when night falls, followed by phosphorylation-dependent activation by cyclic-AMP-dependent activation of protein kinase A (PKA), and protection by binding to 14-3-3 proteins, resulting in increased melatonin production. In contrast, light during the daytime disrupts the AANAT/14-3-3 complex, leading to proteolytic degradation of the enzyme and suppression of melatonin synthesis (Coon et al., 2001; Falcón et al., 2010; Zhang L. et al., 2021).

This review summarizes the evolution and functions of AANAT in various fishes in a genomic perspective. In more detail, we first discuss the origin and copy number variations (CNVs) of *aanat* genes among various species. Next, AANAT sequence diversity and evolution in fish are presented. We further introduce its transcription regulations and expression patterns in several representative species. At last, we review its important physiological roles (mainly through melatonin), especially potential new roles of newly evolved *aanat* duplicates due to gene duplication or whole genome duplication (WGD) to see whether they are shared by teleosts or have occurred independently in specific lineages (Zilberman-Peled et al., 2006).

## Origin of *aanat* Genes in Vertebrates Including Fish

AANAT is classified into two subfamilies, termed non-vertebrate (NV-) AANAT and vertebrate (VT-) AANAT (Falcón et al., 2014); that is to say, NV-AANAT is distributed in non-vertebrate species including cephalochordates, some lower plants and bacteria, while VT-AANAT has been identified in vertebrates such as fish and tetrapods. It is estimated that VT-AANAT evolved from the ancestral NV-AANAT (Falcón et al., 2014). However, these two groups of AANAT proteins show dramatic differences in regulatory and catalytic regions, thus playing totally different metabolic roles (Coon and Klein, 2006; Klein, 2006). This is consistent with a previous hypothesis that vertebrate AANAT was acquired by horizontal gene transfer (Iyer et al., 2004). The questions of when and how VT-AANAT evolved in vertebrates were partly addressed by the identification of the gene in representatives of early divergent vertebrates. First, NV-AANAT had been identified in Cephalochordata (Pavlicek et al., 2010). Then, the first appearance of VT-AANAT was in early vertebrate lineages, in both Agnathans (jawless fish) and Chondrichthyes (cartilaginous fish), suggesting a duplication of the ancestral NV-AANAT through vertebrate genome duplication (VGD) (Falcón et al., 2014). Subsequently, Agnatha and Teleostomi (jawed vertebrates) lost NV-AANAT independently, with only VT-AANAT in these groups (Falcón et al., 2014). However, some taxa within Chondrichthyes keep both NV- and VT-AANATs (Falcón et al., 2014). These findings strongly support the origin of VT-AANAT from the ancestral NV-AANAT. Since the most recent common ancestor of Agnathans and Chondrichthyes dated back to 500 million years ago (Mya) (Inoue et al., 2010), it is estimated that VT-AANAT in vertebrates may originate in the early Cambrian period, before or concomitant with the emergence of lateral eyes and the pineal gland (Falcón et al., 2014).

Because of the two rounds of VGDs shared among vertebrates, the ancestral vertebrates evolved into two copies of *aanat* genes named VT-*aanat1* and VT-*aanat2* (Falcón et al., 2014). However, only one of the duplicates was retained in tetrapods, while cases in fish are more complicated. Besides the VGDs, fish have an additional teleost-specific genome duplication (TGD, third-round WGD, or 3R) that occurred at the root of the teleost lineage at 320 Mya (Opazo et al., 2012; Glasauer and Neuhauss, 2014). Therefore, while losing the NV-AANAT and retaining both VT-AANATs, teleosts generated another copy of VT-*aanat1* but kept only one VT-*aanat2*, resulting in three VT-*aanat* genes (namely VT-*aanat1a*, VT-*aanat1b*, and VT-*aanat2*) in most diploid fish (Li et al., 2015).

Moreover, some fish groups such as sturgeons (Cheng et al., 2019; Cheng et al., 2020), carps (Xu et al., 2014), and salmons (Lien et al., 2016), are prone to polyploidization, which means that they have experienced the fourth or more rounds of WGD (Zhou and Gui, 2017). Lineage-specific WGDs in these groups provide new genetic resources to generate new VT-AANAT duplicates. We (Li et al., 2015) found that tetraploid Atlantic salmon (*Salmo salar*) owns two VT-*aanat1b* (termed VT-*aanat1b1* and VT-*aanat1b2*) and two VT-*aanat2* (termed VT-*aanat2a* and VT-*aanat2b*). So does the tetraploid rainbow trout (*Oncorhynchus mykiss*) (Li et al., 2015). Nevertheless, no comprehensive study has been conducted on such a topic so far. It is predictable that the origins of each of the newly evolved genes could date back to the time of each independent WGD that occurred in the corresponding group or species. Another way for the origin of new fish *aanat* genes is through tandem gene duplication, which is the case for *aanat2* in Amazon molly (Li et al., 2015), but this is the only report to date.

## Copy Number Variations of Fish *aanat* Genes

**FIGURE 2:**
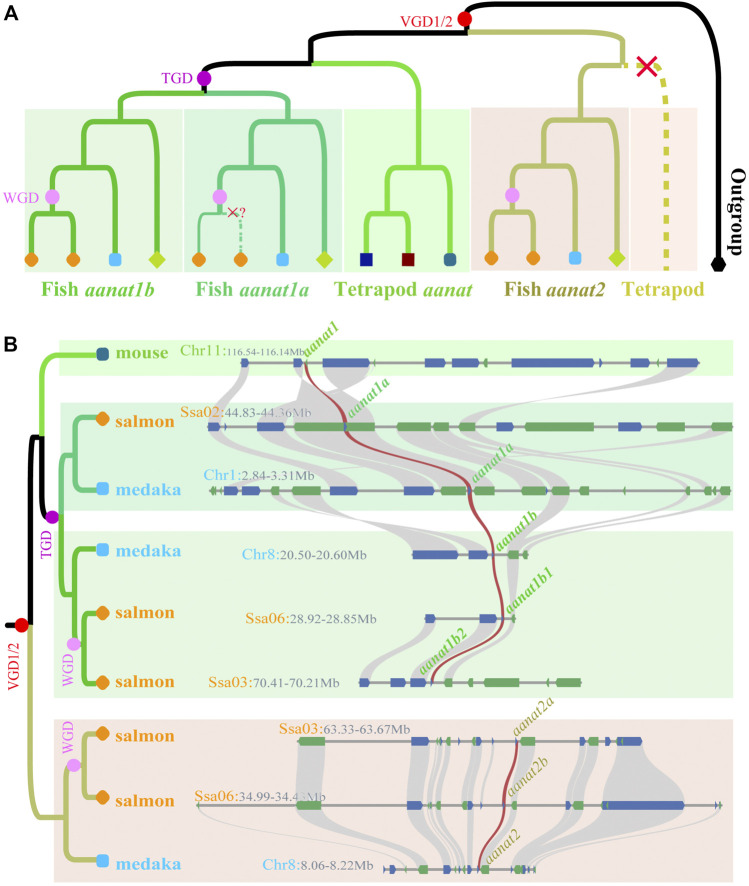
Phylogenetics of fish *aanat* genes. **(A)** The phylogenetic model of *aanats*. *aanat* genes from the same species are marked as the same color and shape. Red, purple, and pink dots represent vertebrate, teleost-specific, and lineage-specific genome duplication events, respectively. Red crosses mark the loss of *aanat* in corresponding clades. **(B)** Syntenic relationship among *aanats* in representative species. Twenty genes at the up-stream and down-stream around *aanats* were chosen to establish the syntenic relationship. Blue (+) and green (-) boxes represent genes with different coding directions, and the brown synteny blocks along various species indicate the *aanat* genes.

As discussed previously, the copy number of *aanat* in fish is closely related to the species ploidy. WGDs increase this number, while subsequent gene loss events decrease the total (Li et al., 2015). For quite a long time, only single *aanat* genes had been identified in birds, reptiles, and mammals except for some lineages in cetartiodacyl clade (Yin D. et al., 2021). Coon et al. (1999) firstly reported two *aanat* genes (*aanat1* and *aanat2*) in fish, and all the previously known AANATs belong to the AANAT1 subfamily (Coon et al., 1999). In 2006, Coon and others performed genome analysis of four teleost species, revealing the presence of three *aanat* genes, corresponding to *aanat1a*, *aanat1b*, and *aanat2* (Coon and Klein, 2006). Since then, along with the big discovery of TGD shared by all teleosts (Taylor et al., 2003), it comes to a consensus that fish have three *aanat* genes (Figure 2; Cazaméa-Catalan et al., 2014).

Afterward, with the dramatic increase in the published number of fish genomes, the *aanat* content in many more species have been studied. In 2015, we conducted a comprehensive analysis of the fish *aanat* genes at a genome level (Li et al., 2015). In this report, a total of 84 *aanat* genes were predicted from 37 vertebrate species, including 24 ray-finned fishes. The copy number of *aanat* genes in all these examined species ranges from two to five (Table 1), but most fishes have the representative number of three (Li et al., 2015).

**TABLE 1:** Copy number variations of *aanat* genes in various fishes.

Order	Scientific name	Common name	Ploidy	*aanat1a*	*aanat1b*	*aanat2*	Total
Chimaeriformes	*Callorhinchus milii*	Elephant shark	2n = 2x	1		0	1^a^
Beloniformes	*Oryzias latipes*	Medaka	2n = 2x	1	1	1	3
Characiformes	*Astyanax mexicanus*	Mexican tetra	2n = 2x	1	0	1	2
Cichliformes	*Oreochromis niloticus*	Tilapia	2n = 2x	1	1	1	3
Cypriniformes	*Sinocyclocheilus anshuiensis*	Golden-line fish (Sa)	2n = 4x	1	0	2	3
	*Sinocyclocheilus grahami*	Golden-line fish (Sg)	2n = 4x	1	0	2	3
	*Sinocyclocheilus rhinocerous*	Golden-line fish (Sr)	2n = 4x	1	0	2	3
	*Danio rerio*	Zebrafish	2n = 2x	1	0	1	2
Cyprinodontiformes	*Poecilia formosa*	Amazon molly	2n = 4x	1	1	2	4
	*Xiphophorus maculatus*	Southern platyfish	2n = 2x	1	1	1	3
Esociformes	*Esox lucius*	Northern pike	2n = 2x	1	1	1	3
Gadiformes	*Gadus morhua*	Atlantic cod	2n = 2x	0	1	1	2
Gobiiformes	*Boleophthalmus pectinirostris*	Blue-spotted mudskipper	2n = 2x	1	1	1	3
	*Periophthalmus magnuspinnatus*	Giant-fin mudskipper	2n = 2x	0	1	1	2
Moronidae	*Dicentrarchus labrax*	European seabass	2n = 2x	1	1	1	3
Osteoglossiformes	*Scleropages formosus*	Golden arowana	2n = 2x	1	1	1	3
Perciformes	*Gasterosteus aculeatus*	Stickleback	2n = 2x	1	1	1	3
	*Epinephelus malabaricus*	Malabar grouper	2n = 2x	1	1	1	3
	*Sparus aurata*	Gilthead seabream	2n = 2x	1	0	1	2
Pleuronectiformes	*Cynoglossus semilaevis*	Tongue sole	2n = 2x	1	1	1	3
Salmoniformes	*Oncorhynchus mykiss*	Rainbow trout	2n = 4x	1	2	2	5
	*Salmo salar*	Atlantic salmon	2n = 4x	1	2	2	5
Syngnathiformes	*Hippocampus comes*	Seahorse	2n = 2x	1	1	1	3
Tetraodontiformes	*Takifugu rubripes*	Fugu	2n = 2x	1	1	1	3
	*Tetraodon nigroviridis*	Spotted green puffer	2n = 2x	1	1	1	3

a Total number of aanat genes refers to VT-aanat; it does not include NV-aanat.

Species with three *aanat* genes are usually diploids, but several other diploid fish have lost one of the *aanat1* genes randomly, reducing the copy number to two. Atlantic cod and Mexican tetra, for instance, have lost *aanat1a* and *aanat1b*, respectively (Li et al., 2015). The loss of *aanat2* has not been identified in any fish genomes so far (Lv et al., 2020), suggesting a highly conserved function of this isotype (Zilberman-Peled et al., 2011).

Several tetraploids, such as the Chinese golden-line fish, also have three *aanat* genes (one *aanat1a* and two *aanat2*). They lost *aanat1b* originated from TGD (Li et al., 2015) and duplicated only *aanat2* during a *Sinocyclocheilus*-specific WGD event (Yang et al., 2016). Another tetraploid, Amazon molly, retains all three *aanat* copies as in diploids, but has evolved into another *aanat2* that was possibly generated by tandem gene duplication instead of WGD, thereby increasing the total number to four. While both rainbow trout and Atlantic salmon have five *aanat* genes because of the salmonid-specific WGD (Ss4R), with one more *aanat1b* than Amazon molly, interestingly, no fish has six or more *aanat* genes to date, but it might be possible in hexaploid and more polyploid fishes such as sturgeons (Ludwig et al., 2001; Cheng et al., 2019) that are under in-depth investigations.

Presumably, CNVs of *aanat* in fish are a combined result of gene duplication and WGD followed by gene loss (Falcón et al., 2014), while some fish or groups have their own preferences on which *aanat* duplicate is to be retained and which one is to be lost. In ray-fins, *aanat2* seems to be essential and is retained in all genomes sequenced so far (Table 1), although it may be inactivated due to frameshift mutations in certain species like deep-sea snailfish (Lv et al., 2020; Mu et al., 2021). Amphibious giant-fin mudskipper has lost *aanat1a* (You et al., 2014), whereas Ostariophysi group has lost *aanat1b* (Li et al., 2015). Such a genetic diversity in various fishes suggests that different isoforms of AANAT may have discrepant functions (Zilberman-Peled et al., 2011).

## Phylogenetic Evolution of *aanat* Genes in Fish

A phylogenetic tree based on coding sequences divides vertebrate *aanat* genes into two main groups by using lamprey (*Petromyzon marinus*) as the outgroup (Figure 2). One group contains tetrapod *aanat* and teleost *aanat1* (*aanat1a* and *aanat1b*), and the other is composed of teleost *aanat2*. Such a consistent topology supports a closer relationship between tetrapod *aanat* and teleost *aanat1*, suggesting that the newly evolved *aanat2* through VGDs in the ancestral vertebrates (Falcón et al., 2014) was lost immediately in tetrapods. In addition, the fish *aanat1* clade was further divided into two groups of *aanat1a* and *aanat1b*, possibly as a consequence of TGD (Li et al., 2015).

After being separated from tetrapod *aanat*, teleost *aanat1* is divided into two subgroups as well, namely *aanat1a* and *aanat1b* (Figure 2). Within each subgroup, the gene tree is usually consistent with the species tree, except for few conflicting nodes. As members in Ostariophysi, Mexican tetra, zebrafish, and three Chinese golden-line barbels have only one *aanat1* closely related to *aanat1b* in the phylogenetic tree, but synteny analysis strongly support the Ostariophysi *aanat1* gene to be *aanat1a* (Li et al., 2015). Similarly, *aanat2* of Ostariophysi, together with the Asian arowana, forms a sister-clade to all other fish *aanat2* genes. This phenomenon of such a special clade formed by Ostariophysi *aanat* genes has been discussed previously (Li et al., 2015), but the detailed reasons and the evolutionary importance behind this phenomenon still needs more investigations.

Fish *aanat* genes and their corresponding paralogs originating from lineage-specific WGDs after TGD always group together, although some nodes are not well resolved. For example, Ss4R occurred in the common ancestor of salmonids nearly 80 Mya, resulting in the acquisition of an additional *aanat1b* in both rainbow trout and Atlantic salmon (Berthelot et al., 2014; Macqueen and Johnston, 2014; Lien et al., 2016). The four *aanat1b* genes form a sister-clade to the pike *aanat1b* as expected, but within this clade, the newly evolved paralogs and the original copies are not sisters to each other. For another instance, six *aanat2* genes (two from each of the three species) diverge to form two clades showing different topologies. One clade consists of the original gene directly descended from a vertebrate ancestor, while the other is duplicated *via* the recent *Sinocyclocheilus*-specific WGD (Yang et al., 2016). With the sequencing of more fish genomes and identification of more *aanat* genes, the evolution of *aanat* in various fishes and its relationship with WGD can be better resolved and illustrated.

An explosive acceleration of evolution has been detected in the stem of the VT-AANAT subfamily after splitting from NV-AANAT, which was presumably associated with the functional shift to melatonin synthesis (Falcón et al., 2014). However, the calculation of evolutionary rates has not yet been applied to fish *aanat* genes specifically. It is still unclear that how fast *aanat* genes have evolved following TGD and multiple rounds of WGD afterward in teleosts.

## AANAT Sequences and Structures

**FIGURE 3:**
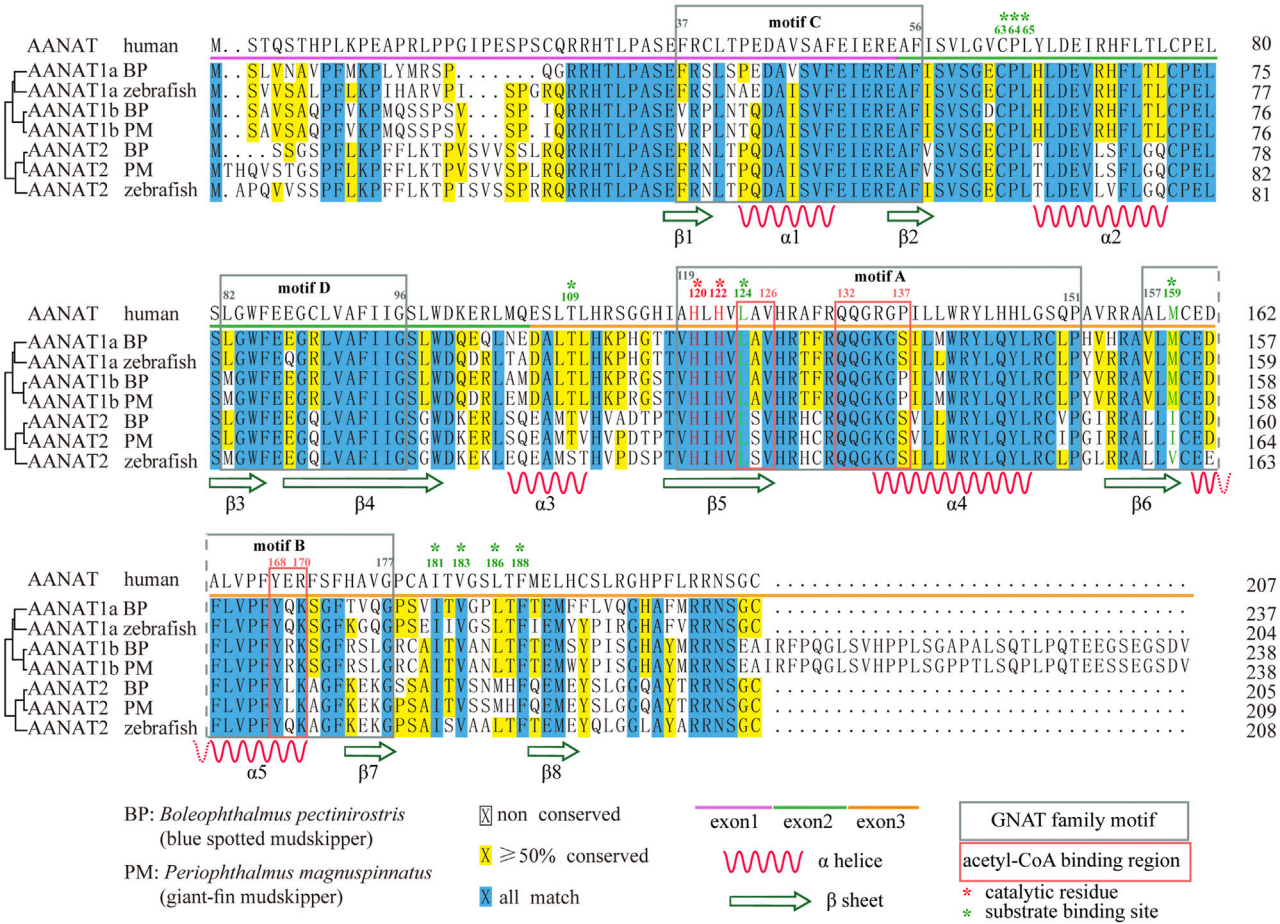
Sequence alignment of AANAT proteins. Conserved motifs and secondary structures of alpha helix (*α*) and beta strand (*β*) are shown. Catalytic residues (red) and substrate binding sites (green) are marked with asterisks. BP (*Boleophthalmus pectinirostris*) and PM (*Periophthalmus magnuspinnatus*) are two representative amphibious mudskippers (You et al., 2014).

Encoding regions of fish *aanat* genes are relatively conserved with high similarities to other vertebrates (Pavlicek et al., 2010; Falcón et al., 2014), consisting of three exons ranging from 150 to 300 bp. The third exon of *aanat1b* is usually longer, increasing the length to over 400 bp. Therefore, the amino acid (aa) sequence-coded by each exon consists of about 50–100 residues individually, while the last exon of AANAT1b encodes 35 more residues (Figure 3).

As a conserved catalytic enzyme, AANAT has a high sequence identity of nearly 60–97% among ten studied vertebrates including sharks, fishes, reptiles, and mammals (Li et al., 2015). Within fish, the identity is even higher. Specifically, fish AANAT1a proteins are the most conserved with a minimum identity of 88%, followed by AANAT2 (81%), while AANAT1b is the most variable (71%) among vertebrate species (Li et al., 2015). Therefore, AANAT proteins have been highly conserved throughout fish evolution, indicating that they have been maintained by natural selection for important biological functions in various fishes (Kuz’mina, 2020).

**FIGURE 4:**
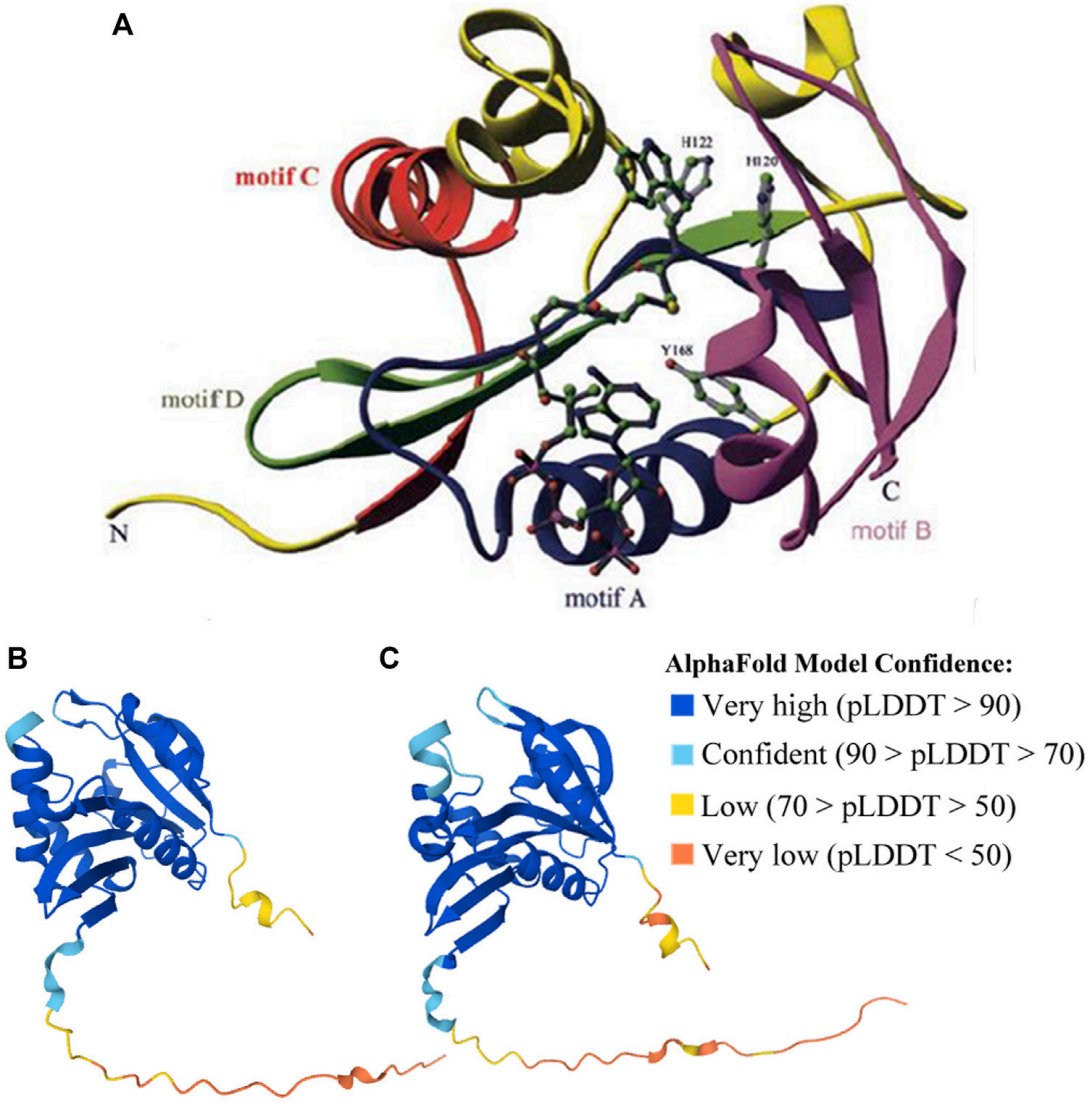
Structures of human (validated) and zebrafish (predicted by AlphaFold) AANATs. **(A)** X-ray structure of human AANAT bound to bisubstrate analog, highlighting the four GNAT family motifs (marked as motifs A–D), and catalytic histidine (H120 and H122) residues. It was adopted from Scheibner et al. (2002). **(B)** Zebrafish AANAT1 (UniProt accession Q6V7J8). **(C)** Zebrafish AANAT2 (UniProt accession Q9PVD7).

AANAT is also structurally conserved (Figure 4). Previous reports have shown that VT-AANAT has a conserved fold consisting of about eight *β* sheets flanked by five *α* helices, and shares four conserved GNAT family motifs designated A-D (Hickman et al., 1999), among which motif B contributes acidic residues to the serotonin binding slot (Wolf et al., 2002). Take the human AANAT (207 aa in length; UniProt accession Q16613; Figure 4A) as an example, there are a conserved *N*-acetyltransferase domain (35–194), three acetyl-CoA binding regions (124–126, 132–137, and 168–170), two catalytic histidine residues (His120 and His122), and multiple substrate binding sites (Leu124 *via* amide nitrogen, and Met159 *via* carbonyloxygen; Figures 3,4) (Scheibner et al., 2002; Wolf et al., 2002).

The structures of fish AANATs predicted by AlphaFold (Jumper et al., 2021) are very similar to human AANAT, especially in those conserved motifs and residues (Figures 3, 4B,C). All examined fishes share with humans the same four GNAT family motifs, the three acetyl-CoA binding regions, the two catalytic histidine sites, and most of the substrate binding sites in their AANATs (Figures 3,4). As shown previously, fish AANAT1 is closer to tetraploid AANAT and has fewer variants in comparison to fish AANAT2. Multiple substrate binding sites (T109, M159, and L196) have been mutated to other amino acids in AANAT2 but remain the same in AANAT1.

Mutations of some amino acid sites have been observed, such as F130C and V153L in AANAT2 (Li et al., 2015). Some mutations are found in both fish and humans. The most interesting one is the alternative of Ala129 to Tyr in AANAT1 and to His in AANAT2. This site has been recognized as a single-nucleotide polymorphism (SNP) in humans, showing that individuals with the natural variant A129T have delayed sleep phase syndrome (Hohjoh et al., 2003). Although the impact of such a mutation in fish has not been examined, it is possible that this change might have something to do with the sleep pattern difference between humans and fish.

## Aanat Expression Patterns

**FIGURE 5:**
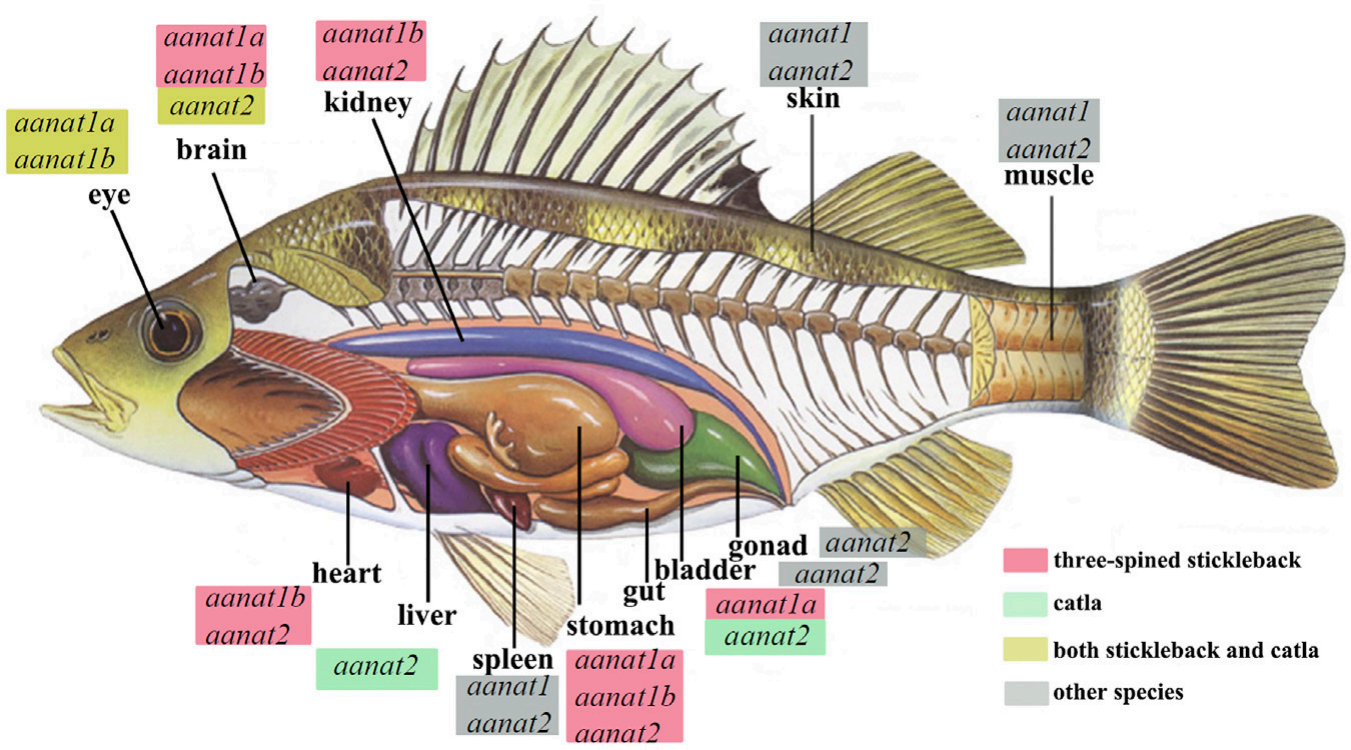
Endogenous sources of melatonin and *aanat* expression in various fishes, including carp (*Catla*; Maitra and Pal, 2017) and three-spined stickleback (*Gasterosteus aculeatus*; Kulczykowska et al., 2017). Detection of *aanat* mRNA in different tissues is according to the references. *aanat* with a red box marks its existence in the corresponding tissue of the stickleback, *aanat* with a green box refers to its existence in the catla carp, while those with a yellow box means *aanat*(s) expressed both in stickleback and catla. “Other species” are the goldlined spinefoot (*Siganus guttatus*), rainbow trout (*Oncorhynchus mykiss*), goldfish (*Carassius auratus*), and sea bass (*Dicentrarchus labrax*) (Kulczykowska et al., 2017).

A newly arisen duplicate of any gene has three possible fates, being lost (including being silenced by degenerative mutations), subfunctionalization or neofunctionalization (Lynch et al., 2001), and the retained duplicate genes may also alter their expression pattern *via* cooperation (True and Carroll, 2002). Multiple *aanat* genes in teleosts displaying different expression patterns (Figure 5) is a good example of such a process.

The single human *aanat* is mainly expressed in the pineal gland and retina (Coon et al., 1996), but it has also been identified in other tissues such as testis (Albrecht, 2009) and skin (Slominski et al., 2002). In fish, the two types of *aanat* display tissue specific distribution. Expression of *aanat1* (both *aanat1a* and *aanat1b*) have been detected mainly in the retina and brain, while *aanat2* is reported to be specifically expressed in the pineal gland, despite that, positive detection of *aanat1a* and *aanat1b* has also been observed in dorsal sac (surrounding the pineal organ) (Isorna et al., 2009; Isorna et al., 2011; Paulin et al., 2015). However, the expression of *aanat* genes has also been detected in other fish tissues, such as gill, kidney, liver, spleen, skin, gonad, and gut (Figure 5) (Fernández-Durán et al., 2007; Velarde et al., 2010; Falcón et al., 2011; Nisembaum et al., 2013; Sanjita Devi et al., 2016; Maitra and Pal, 2017; Kulczykowska et al., 2017; Kuz’mina, 2020).

**FIGURE 6:**
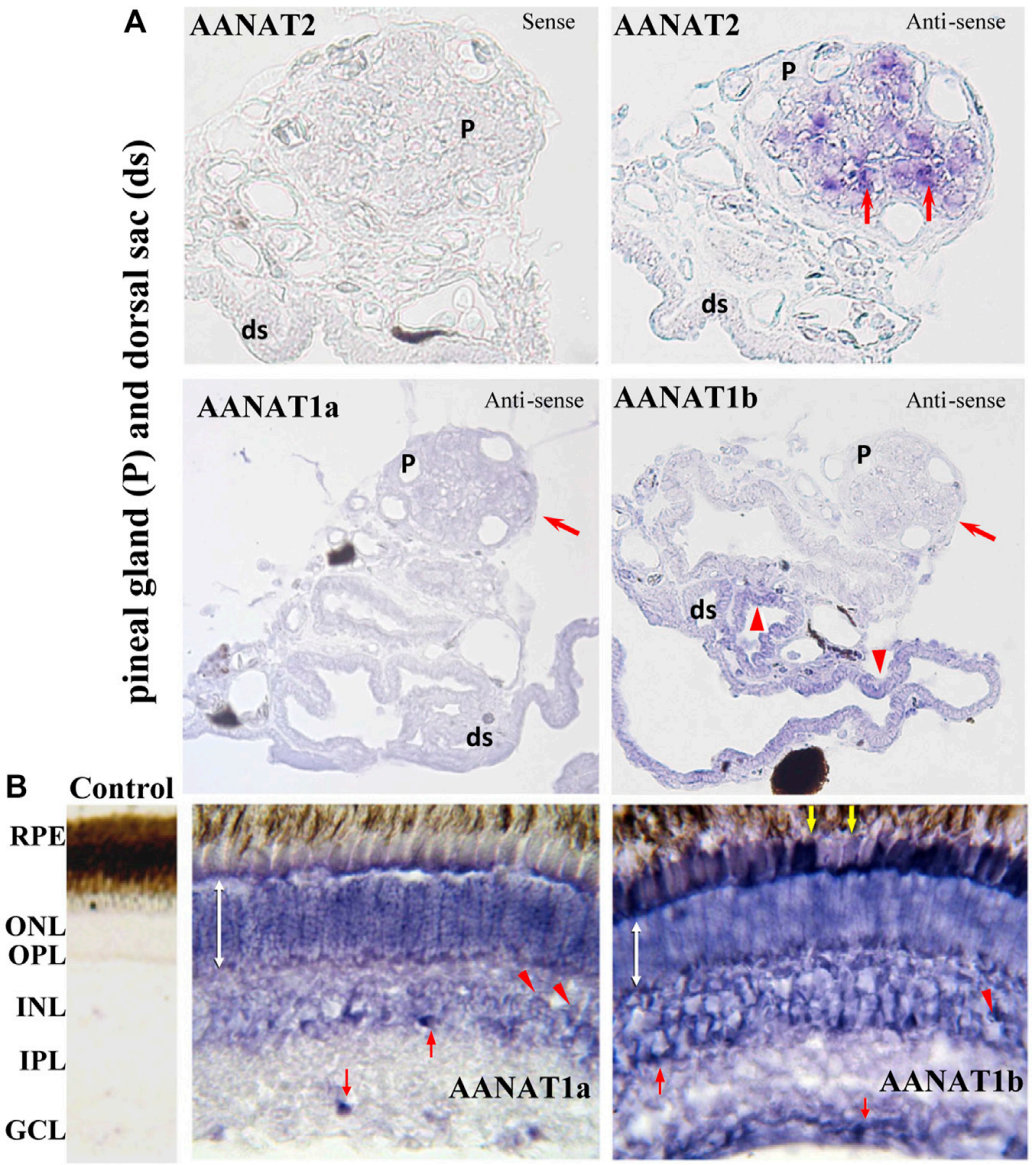
Detection of *aanat* (*aanat1a*, *aanat1b,* and *aanat2*) transcriptions in fish pineal gland **(A)** and retina **(B)** (adopted from Paulin et al., 2015). **(A)** Pinealocytes (arrows) are labeled with the antisense *aanat2* probe, but not with either *aanat1a* or *aanat1b* probes. Cells of dorsal sac (arrow heads) are stained with *aanat1b* antisense probes but not with *aanat2* probe. ds: dorsal sac; P: pineal gland. **(B)** Photoreceptor cells of the ONL are stained (double white arrow). Bipolar (arrow heads) and amacrine (upward red arrow) cells in the INL are also labeled. A few cells are labeled in the GCL (downward red arrow) and RPE (downward yellow arrows). GCL: ganglion cell layer; INL: inner nuclear layer; IPL: inner plexiform layer; ONL: outer nuclear layer; OPL: outer plexiform layer; RPE: retinal pigment epithelium.

Although both *aanat1a* and *aanat1b* are mainly expressed in retina and brain, their abundance, day-night or lifespan expression patterns have been reported to be distinctly different (Isorna et al., 2011). In flatfish (*Solea senegalensis*), *aanat1a* has a lower expression level than *aanat1b* in the retina of adults. Expression of *aanat1a* was more abundant during the early than late larval stages, while *aanat1b* expression was low during early developing stages but rose significantly throughout metamorphosis (Isorna et al., 2011). Furthermore, the expression levels of the two *aanat1* genes also differ in outer nuclear (ONL) and inner nuclear (INL) layers of retina, and in the ganglion cell layers (GCL) (Isorna et al., 2011; Paulin et al., 2015) (Figure 6B).

The notable differences of expression patterns in the tissue distribution, abundance, and the day-night rhythm during development stages throughout their lifespan suggest different functions for the three fish AANAT enzymes (Klein, 2007; Paulin et al., 2015). Further investigations are needed to pay attention to their functional characteristics, and to elucidate their biological roles in regulating the biosynthesis of melatonin, dopamine, and other potential metabolites.

## Various Functions of AANAT in Fish

Where and how the three fish AANAT enzymes being expressed strongly suggest that they have various functions. Pineal AANAT2 functions mainly in the melatonin biosynthesis and prefers indolylethylamine over phenylethylamine as its substrate (Paulin et al., 2015). It always follows a day–night rhythm (Zilberman-Peled et al., 2007) and sometimes exhibits a temperature dependency (Cazaméa-Catalan et al., 2012; Paulin et al., 2015). However, the role of retina AANAT1s is less apparent. Having similar affinity for both phenylethylamines and indolylethylamines, AANAT1 enzymes seem to have a broader range of functions in addition to catalyzing the synthesis of melatonin as AANAT2 does. They are also reported to be involved in the catabolism of serotonin and dopamine (Falcón et al., 2010; Paulin et al., 2015; Maitra and Pal, 2017).

There are accumulated proofs that fish AANAT enzymes have more functions than just being the “timezyme” for synthesizing the time-measuring hormone melatonin (Klein, 2007). More roles such as detoxification (Besseau et al., 2006; Zilberman-Peled et al., 2011) and neurotransmission (Zilberman-Peled et al., 2006) have already been discussed before, and new functions might be discovered with increasing fish genomic data (Sun et al., 2016; Fan et al., 2020) and verification experiments, especially in fishes with multiple copies of AANATs (Li et al., 2015). More studies are necessary to determine why some fish keep more AANATs while other species lose one or more *aanat* genes (Table 1). Answers to what roles the additional AANATs play and what impacts the gene loss will be valuable for an in-depth understanding of fish AANATs. Here, we provide a summary of multiple important physiological roles of AANATs and melatonin in various fishes.

### Seasonal Migration

Juvenile chum salmons (*Oncorhynchus keta*) in the rivers of Hokkaido in Northern Japan usually initiate their catadromous migration around March to April each year (at about 100 days after hatching), when the river and shallow-sea surface ices are almost melted. In 2004, we cloned two *aanat*s (*aanat1* and *aanat2*) and two melatonin receptor genes (*mel1a* and *mel1b*) in chum salmon, and measured melatonin levels as well as mRNA levels of the four genes in the eye and brain during embryonic and post-embryonic stages (Shi et al., 2004).

**FIGURE 7:**
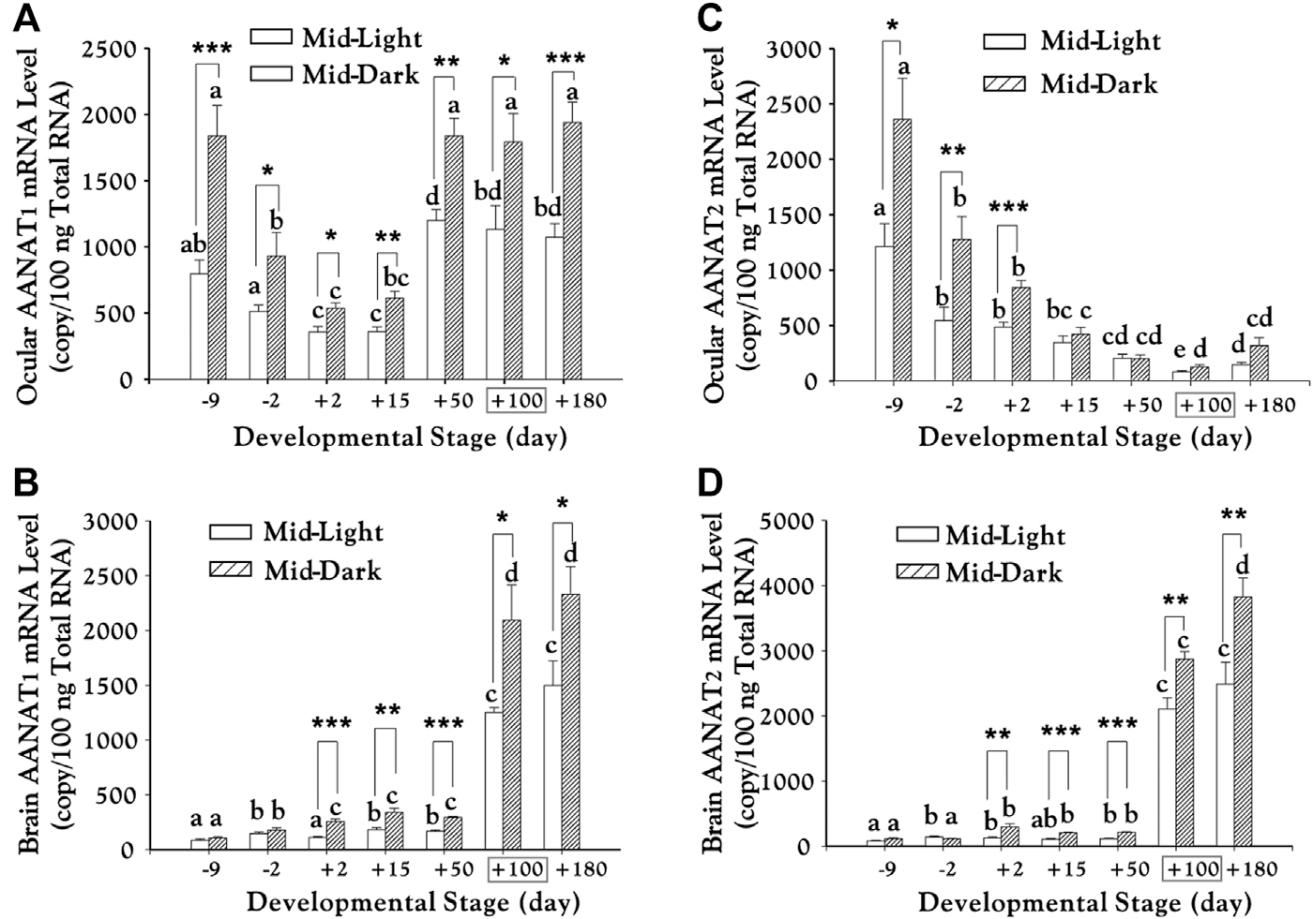
Transcription of *aanat1* and *aanat2* mRNAs in the eye **(A–C)** and brain **(B–D)** of chum salmon (adopted from Shi et al., 2004). Juveniles begin to migrate downstream from day +100 (boxed). Duncan’s multiple range test (different letters show *p* < 0.05) and Student’s t test (*, *p* < 0.05; **, *p* < 0.01; ***, and *p* < 0.001) were used to compare the significance between mid-light or mid-dark values at different developmental stages and within the same date, respectively.

The study showed that shortly before the spring season, *aanat* mRNAs and melatonin levels in the eye and brain of these pre-spawning chum salmons had been elevated to peak values (Figure 7), suggesting that these parameters are important signals for seasonal migration of chum salmon (Shi et al., 2004). The obvious parallelism in developmental changes and circadian rhythms of *aanat* mRNAs and melatonin levels supports the popular hypothesis that the developmental increases of nocturnal melatonin levels are in part a consequence of the elevated transcription of pineal *aanat* genes (Shi et al., 2004; Shi, 2005).

Moreover, abundant *aanat* and *mel-R* mRNAs in various brain regions and eyes indicates potential roles of melatonin in visual processing and neuroendocrine regulation, through which melatonin might be involved in migratory behaviors of chum salmon (Shi et al., 2004). Interestingly, this report tried to separate *aanat* into *aanat*-pineal and *aanat*-retina, which were renamed formally as *aanat1a* and *aanat1b* since 2006 (Coon and Klein, 2006).

### Amphibious Vision

Mudskippers are the biggest group of amphibious fishes with a division of four main genera, including *Boleophthalmus*, *Periophthalmodon*, *Periophthalmus*, and *Scartelaos*. They represent a continuum of adaptations toward terrestrial life, with some species being more terrestrial than the others. In 2014, we published the first genome article (You et al., 2014) of the four representative species of mudskippers, mainly focusing on the blue spotted mudskipper (*B. pectinirostris*; BP) and giant-fin mudskipper (*Periophthalmus magnuspinnatus*; PM). Comparative genomics analyses were performed to provide novel insights into the genetic basis of terrestrial adaptation in these mudskippers.

Usually, fully aquatic fishes are likely to somehow have myopic vision in an air condition (You et al., 2014; You et al., 2018). However, mudskippers seem to have good aerial vision due to their strong ability to avoid terrestrial predators. Comparison of vision-related genes in the two representative mudskippers (BP and PM) and several vertebrates highlighted certain adaptive losses or mutations in mudskippers (You et al., 2014).

With the detailed genomic data, we noted that BP contains all the three *aanat* genes whereas PM possesses only *aanat1b* and *aanat2*. The existence of *aanat1b* was confirmed by abundant reads mapping to the gene locus. In contrast, no PM reads could be mapped to the *aanat1a* sequence of BP, suggesting that PM may have lost the *aanat1a* gene. In fact, dopamine acetylation is a novel function of fish AANAT1a in retinae (Zilberman-Peled et al., 2006), which has been proposed to cause low retinal-dopamine levels progressively leading to myopia (Feldkaemper and Schaeffel, 2013). We therefore speculate that the loss of *aanat1a* in PM may have generated an elevation status of retina dopamine levels in the occurrence of myopia, which would facilitate aerial vision for a selective advantage in PM so as to spend most (over two-third) of its lifetime on an intertidal mudflat surface (You et al., 2014; You et al., 2018).

### Cave or Deep-Sea Adaptation

Cavefishes have often developed degenerated features, such as rudimentary eyes or scales, and loss of pigmentation. As potential compensation, some more sensitive traits have evolved in these fish, such as elongated appendages and non-visual positioning or sensory systems (Yang et al., 2016).

*Sinocyclocheilus* is endemic to China’s Qinghai Tibetan Plateau. This genus of over 75 species is a good cavefish model due to its high species diversity and phenotypic variations. In 2013, a Science letter reported over 150 naturally caved species in southwestern China, which were unknown before (Shu et al., 2013). Subsequently, genomes of three *Sinocyclocheilus* species were published (Yang et al., 2016; Yin Y. H. et al., 2021), including surface-dwelling *S. grahami* (Sg), semi-cave-dwelling *S. rhinocerous* (Sr), and cave-restricted *S. anshuiensis* (Sa). They are representatives of three key nodes on the path to a cave life. Interestingly, Sa has sometimes lost its external eyeballs and lens, and its body has become somehow transparent or with albinism. Although abnormality of *aanat* genes has not been determined in these species, we identified a similar premature stop in the encoding region of *aaad* gene as we found in the cave-restricted Mexican tetra (Lv et al., 2020), implying a possibility of weakening or disappearing rhythms in cavefishes, possibly caused by low melatonin levels (Yang et al., 2016).

Recently, a draft genome of *Pseudoliparis swirei*, a deep-sea snailfish (Mariana hadal snailfish, MHS) with a routine residence below 6,000 m, was published (Wang et al., 2019). Meanwhile, the genome of its closed relative Tanaka’s snailfish (*Liparis tanakae*, TS) from shallow sea was also available in the same article. We performed a detailed comparison of *aanat2* gene structures between MHS and TS (Lv et al., 2020), and observed a frameshift insertion in MHS, while its relative TS was normal. As we supposed, the insertion may lead to an inactivity of AANAT2, and hence consequent low levels of blood melatonin in MHS (Lv et al., 2020). These are possibly related to the deep-sea darkness adaption, which is similar to our previously reported cave-restricted Sa (Yang et al., 2016) for cave adaptation.

More recently, we observed the similarly potential inactivation of AANAT2 in a Yap hadal snailfish (YHS) (Mu et al., 2021), which was collected at a 6,903 m depth. These results suggest that the low visual ability of MHS/YHS (Wang et al., 2019; Mu et al., 2021) may be similar to the cavefish-like degenerated eyes with a principal sense of shortwave light (You et al., 2018). Therefore, reducing melatonin synthesis by inactivation of AANAT or other melatonin biosynthesizing enzyme(s) may be a common mechanism for cave or deep-sea adaptation (Lv et al., 2020).

### Other Melatonin Effects

In addition to the aforementioned functions of AANATs in fish, most studies emphasized melatonin effects in the regulation of the following biological processes.

First, the impact of melatonin on the seasonal cycle of fish reproduction (Kumar Bairwa et al., 2013; Maitra and Hasan, 2016) has been largely investigated. The first report was in 1996 focusing on Atlantic croaker (*Micropogonias undulates*) (Khan and Thomas, 1996), followed by increasing number of reports on other species including stickleback (Kulczykowska et al., 2017), catfishes (Chaube and Joy, 2002; Martinez-Chavez et al., 2008; Aripin et al., 2015; Badruzzaman et al., 2020), and a non-air–breathing subtropical carp (*Catla*) being extensively used as a model to study fish melatonin (Figure 5) (Bhattacharya et al., 2007; Moniruzzaman and Maitra, 2012; Maitra et al., 2013; Hasan et al., 2014). It is now believed that melatonin not only acts as a hormone in determining the temporal pattern of spawning, but also as an antioxidant in regulation of oocyte maturation at the downstream of hypothalamus-pituitary-gonad (HPG) axis in fish (Shi, 2005; Maitra and Hasan, 2016).

Secondly, melatonin also impacts food intake and growth, which is largely dependent on day-length. Melatonin produced in pineal gland has been proved to control the related behavioral rhythms (Zhdanova et al., 2001; Falcón et al., 2010). However, various experimental results in different species (Spieler, 2001; Taylor et al., 2005; De Pedro et al., 2008) have not drawn a unanimous conclusion of exactly how melatonin impacts growth. There are estimations that melatonin may work by regulating the release of growth hormone (GH), plasma prolactin (PRL), and perhaps other pituitary hormones (Falcón et al., 2010; Falcón et al., 2011).

Additional functions of melatonin in fish include its influences on the water–salt balance, regulation of the antioxidant system, involvement in the immune system, and so on (Kuz’mina, 2020). However, the molecular mechanisms underlying each of these broad functions have not been fully elucidated yet.

## Conclusions and Perspectives

Fish have the most diversity of *aanat* genes (*aanat1a*, *aanat1b,* and *aanat2*) in vertebrates (Li et al., 2015). AANAT plays critical roles in melatonin biosynthesis, and sometimes for potential dopamine metabolism, which are responsible for various physiological functions, such as seasonal migration, amphibious aerial vision, and cave or deep-sea adaptation. Interestingly, many transparent fish (Liu et al., 2017; Bian et al., 2020) are short of melatonin, possibly due to an inactivation of AANAT or other melatonin biosynthesizing enzyme(s); their shorter life-time (half a year for a transparent *roy* zebrafish (Bian et al., 2020), instead of 3–5 years for the wildtype) suggests that restoration of AANAT or melatonin may become an effective way to increase the lifetime of transparent fish. Meanwhile, accumulated genome and transcriptome data (Li et al., 2015; Sun et al., 2016; Hughes et al., 2018) provide genetic resources to obtain more and more sequences of *aanat* genes, which will enrich our understanding of AANAT and melatonin functions in various fishes, and ultimately improve and standardize husbandry practices (such as management of light, feeding, and spawning) in fish aquaculture worldwide.

## Abbreviations

AAAD, aromatic *L*-amino acid decarboxylase; AANAT, SNAT or NAT, arylalkylamine *N*-acetyltransferase; ASMT, acetylserotonin-*O*-methyltransferase; CNV, copy number variation; GCL, ganglion cell layer; GCN5, general control non-repressed five protein; GNAT, GCN5-related *N*-acetyltransferase; 5-HT, serotonin; HTrp, 5-hydroxytryptophan; INL, inner nuclear layer; NAS, *N*-acetylserotonin; NV-AANAT, nonvertebrate AANAT; ONL, outer nuclear layer; Ss4R, salmonid-specific WGD; TGD, teleost-specific genome duplication; TPH, tryptophan hydroxylase; Trp, tryptophan; VGD, vertebrate genome duplication; VT-AANAT, vertebrate AANAT; WGD, whole genome duplication.
